# Structure-based discovery of a novel Granzyme A (GZMA) inhibitor for autism spectrum disorder through integrative transcriptomics, virtual screening, and long-timescale molecular dynamics simulations

**DOI:** 10.1371/journal.pone.0359103

**Published:** 2026-10-13

**Authors:** Metab Alharbi, Abdulrahman Alshammari, Othman Al Shabanah, Mohammed Mufadhe Alanazi, Ahmed Altuwaijri, Abdullah F. Alasmari, Talal Alshammari

**Affiliations:** Department of Pharmacology and Toxicology, College of Pharmacy, King Saud University, Riyadh, Saudi Arabia; DAI Fleming Fund Pakistan, PAKISTAN

## Abstract

Granzyme A (GZMA) is a trypsin-like serine protease expressed in cytotoxic lymphocytes that cleaves gasdermin B (GSDMB) to release a pore-forming N-terminal fragment, thereby inducing pyroptosis, a lytic and pro-inflammatory form of programmed cell death. This pathway has been implicated in immune dysregulation reported in a subset of individuals with autism spectrum disorder (ASD). In this study, we reanalyzed the peripheral blood transcriptome dataset GSE26415 using an integrative computational framework comprising outlier-aware quality control, differential expression analysis, functional enrichment profiling, weighted gene co-expression network analysis, and protein–protein interaction network centrality scoring. These orthogonal evidence layers were integrated into a composite prioritization score, in which GZMA ranked first among candidate genes, followed by KLRK1 and KLRB1. Querying the Drug-Gene Interaction Database revealed no approved small-molecule inhibitors targeting GZMA, indicating that it remains pharmacologically undrugged. To explore druggability at the structural level, we performed structure-based virtual screening of a ZINC similarity library against the active site of GZMA (PDB ID: 1ORF). This yielded three drug-like candidates (T1, T2, and T3) with docking scores of −9.3, −9.1, and −8.9 kcal/mol, respectively, compared with −7.4 kcal/mol for the co-crystallized reference ligand (0G6). Each drug-protein complex and apo enzyme was studied in 500 ns molecular dynamics to verify the binding stability and conformational persistence compared to the unliganded protein. Among the screening compounds, T2 was able to occupy the catalytic pocket for the entire simulation, with a stable protein-ligand interaction and one low-energy conformational basin. T1, despite a better docking score, was able to detach from the pocket after around 400 ns, while T3 was able to do so in 50 ns. The overall order of binding persistence was T2 > T1 > T3. Collectively, these results prioritize GZMA as a key ASD-associated immune effector candidate and identify T2 as the most credible lead compound. Importantly, the findings underscore the limitation of docking scores alone and demonstrate that long-timescale molecular dynamics simulations and binding free energy are essential for distinguishing kinetically stable binders from transient docking poses. We note that GZMA was transcriptionally downregulated in peripheral blood; its selection rests on network centrality and on the premise that granzyme pathology is governed by enzymatic activity at tissue sites rather than by circulating transcript abundance, a mechanistic hypothesis to be tested at the activity level.

## 1. Introduction

Autism spectrum disorder (ASD) [1] is a neurodevelopmental disorder with persistent difficulty in social communication and interaction, and restricted and repetitive patterns of behavior [2]. The most recent surveillance of the U.S. Autism and Developmental Disabilities Monitoring Network estimated the prevalence of ASD at approximately 1 in 31 children aged 8 years during the 2022 surveillance year, and 1 in 36 in the 2020 surveillance year [3]. The biological origin of ASD is quite diverse, and the most likely explanation is genetic. In recent years, the immune system dysregulation in some people with ASD has become a clinical reality; studies have found the activation of peripheral myeloid cells, pro-inflammatory cytokines, and microglial activation in the brain tissue, and the extent of these abnormalities is associated with behavioral symptoms. [4,5]. Because brain tissue is not generally accessible to humans while alive, the use of peripheral blood transcriptomics is a promising tool in the search for molecular changes associated with ASD, as well as potential biomarkers and treatment targets. Several transcriptomic studies have uncovered differences in gene expression in peripheral blood leukocytes between people with ASD and typical neurotypical populations. The GSE26415 dataset of Kuwano et al. was among those. This dataset revealed an ASD-associated transcriptional signature in venous leukocytes, which was also observed in unaffected mothers of children with ASD [1].

Granzyme A (GZMA) is a known immune agent that may have a mechanistic link between immune dysregulation and inflammatory cell death. GZMA is one of the most abundant serine proteases in the cytotoxic granules of activated T lymphocytes and natural killer cells [6]. While GZMA is known to be a cytotoxic mediator, it has an important extracellular pro-inflammatory function. In particular, GZMA cleaves gasdermin B (GSDMB) at its interdomain linker (Lys244) and releases a pore-forming fragment that promotes pyroptotic cell death [7]. Structural studies have revealed how this liberated GSDMB fragment is deposited in the membrane pores and how this process is regulated. The GZMA-GSDMB axis is thus becoming more and more recognized as an important mechanism to link cytotoxic lymphocyte activity to pyroptosis and inflammation [8,9]. Importantly, experimental inhibition or inactivation of GZMA has been shown to suppress GSDMB-mediated pyroptosis and inflammation in disease models, and this is a basis for studying GZMA as a therapeutic target [10]. The persistent activation of pyroptotic and inflammatory signaling could be related to the gastrointestinal and systemic immune abnormalities reported in children with ASD and ileocolitis. In these patients, intestinal mucosal transcriptional profiles are similar to those in inflammatory bowel disease but also show features that are associated with ASD-associated gastrointestinal pathology. [11]. If the dysregulated GZMA activity is considered to be responsible for this inflammatory state, then pharmacological inhibition of GZMA could be an effective treatment. GZMA activation upstream of pore formation is particularly promising as it can prevent the release of inflammatory mediators (i.e., interleukin-1β, interleukin-18, and high-mobility group box 1 (HMGB1)) associated with pyroptotic cell death from the downstream mediators in a synchronized manner.

This study identifies candidate small-molecule inhibitors of GZMA through an integrated computational pipeline. We first reanalyzed the GSE26415 microarray data, combining outlier detection, differential expression, functional enrichment, and co-expression networks with protein-protein interaction centrality to prioritize pathological targets, a procedure that placed GZMA first. We then performed structure-based virtual screening against a ZINC similarity library and carried the highest-scoring drug-like compounds into 500 ns molecular dynamics simulations, benchmarked against the apo protein, to test whether the docked poses persist under explicit-solvent dynamics.

## 2. Materials and methods

### 2.1. Microarray data acquisition and quality control

Gene expression data (GSE26415) were retrieved from the NCBI Gene Expression Omnibus [12]. The dataset comprises peripheral-blood leukocyte transcriptomes from individuals with ASD and unaffected controls, profiled on the Agilent GPL6480 platform. Outliers were screened with three complementary metrics: the interquartile range of median log2-transformed intensities, the deviation of each sample's Spearman correlation from the group mean, and a Mahalanobis distance computed from a robust principal component analysis. Samples flagged by any one of these metrics were removed before normalization. A second outlier screen was also applied after surrogate-variable estimation, in which samples falling outside the 95 percent confidence ellipse of the first two principal components of the batch-corrected expression matrix were also excluded.

### 2.2. Differential expression analysis

Statistical modeling was performed in R with the limma package [13]. The vooma function estimated precision weights to accommodate the mean-variance relationship of microarray intensities without imposing quantile normalization, and standard errors were moderated with an empirical Bayes procedure. Differentially expressed genes were defined by a false-discovery-rate-adjusted p-value below 0.05 and an absolute log2 fold change of at least 0.65. This threshold corresponds to a 1.57-fold change and was adopted as a moderately conservative alternative to the conventional 1.5-fold cutoff (log2 = 0.585) commonly applied to whole-blood microarray data, in which true effect sizes are attenuated by cellular heterogeneity. Generic structural genes were filtered using Gene Ontology annotations from the GO.db and org.Hs.e.g.,db databases [14].

### 2.3. Functional enrichment and co-expression networks

Functional profiling of the differentially expressed genes covered Gene Ontology biological processes, KEGG pathways, and gene set enrichment against the Hallmark collection, all performed with clusterProfiler [15]. Transcription-factor activity was inferred with the VIPER algorithm applied to the DoRothEA regulon collection [16,17]. Weighted gene co-expression network analysis built a signed network of correlated modules [18], and module eigengenes were correlated with disease status. Circulating immune-cell composition was estimated from the pre-batch-correction expression matrix using MCP-counter as implemented in the immunedeconv package, and cell-type abundances were compared between groups by two-sided Wilcoxon rank-sum tests with Benjamini-Hochberg correction. To test whether differential GZMA expression was attributable to cell-composition shifts, the group effect on GZMA was re-estimated in a linear model additionally adjusted for the estimated natural killer and CD8 + T-cell abundances.

### 2.4. Protein-protein interaction network and target prioritization

Significant differentially expressed genes were mapped to the STRING database, version 12.0, and the resulting protein-protein interaction network was analyzed in igraph, with hub genes identified by degree, betweenness, closeness, and eigenvector centrality [19]. Candidate targets were ranked by a composite score that weighted interaction degree, interaction betweenness, and absolute log2 fold change at 40, 30, and 30 percent, respectively, with each component min-max normalized before weighting. Known drug-gene interactions were retrieved through the DGIdb GraphQL application programming interface [20].

### 2.5. Protein preparation and virtual screening

The crystal structure of human GZMA in complex with the co-crystal ligand 0G6 (PDB 1ORF)[21]was retrieved from the Protein Data Bank to define the ligand, and a second GZMA structure (PDB 1OP8)[22] was used as the receptor. Two structures were required because the only GZMA structure carrying a co-crystallized active-site inhibitor, 1ORF, is not the wild-type enzyme: it contains an engineered point substitution of Glu for Asn (Asn170 of UniProt P12544). We therefore took the ligand 0G6 and its reference binding pose from 1ORF, which also defines the query for the similarity search, but carried out all docking against the wild-type receptor provided by 1OP8. The two structures are conformationally equivalent over the region relevant to docking: superposition of the matched protomers gives a Ca root-mean-square deviation of 0.60 A over 199 residues, 0.34 A across the backbone of the catalytic triad (His57, Asp102, Ser195), and 0.44 A across the backbone of the 22 residues lining the 0G6 site, with no active-site residue deviating by more than 1.0 A. Cross-structure docking is therefore justified. Water molecules and heteroatoms were removed, hydrogens were added, and Kollman charges were assigned. A similarity search seeded with 0G6 returned 5,309 structurally related compounds from the ZINC database (REALDB-25Q3) accessed on May 20, 2026 [23]. These were docked into the GZMA active site with AutoDock Vina [24], and the three compounds with the most favorable binding affinities were carried forward as test ligands T1 to T3.

### 2.6. Molecular dynamics simulations

Four systems were prepared: the three top-hit GZMA-ligand complexes and the apo protein. Topologies were generated with CHARMM-GUI [25] using the CHARMM36m protein force field [26], with ligand parameters assigned by the CHARMM General Force Field server [27]. Each system was solvated in a cubic TIP3P water box and neutralized with 0.15 M KCl. Energy minimization was performed using steepest descent, followed by a four-stage equilibration totaling 4 ns with a 1 fs time step, in which harmonic position restraints were released stepwise from 400 to 50 kJ mol-1 nm-2 on backbone atoms and from 40 to 5 kJ mol-1 nm-2 on side-chain atoms, under the NVT ensemble for the first 2 ns and the NPT ensemble thereafter with a C-rescale barostat at 1 bar. Potential energy and system density were written every 1 ps and were monitored across the equilibration window. Production runs of 500 ns were carried out for each system in GROMACS 2025 [28,29] with GPU-accelerated particle-mesh Ewald electrostatics, a 2 fs time step, and a temperature of 303.15 K.

### 2.7. Trajectory analysis

Backbone root-mean-square deviation, per-residue root-mean-square fluctuation, and radius of gyration were computed with the GROMACS analysis suite. Ligand stability was assessed in two ways: by fitting on the ligand to report internal flexibility, and by fitting on the protein backbone to report movement of the ligand relative to the pocket. Protein-ligand hydrogen-bond occupancy was assessed using a donor-acceptor distance cutoff of 0.35 nm and an angle cutoff of 30 degrees. Short-range protein-ligand interaction energies were decomposed into Lennard-Jones and Coulomb terms from the GROMACS energy output, and binding-pocket occupancy was quantified as the number of pocket-ligand atom pairs within 0.35 nm together with the pocket-localized hydrogen-bond count. Principal component analysis and free energy surfaces were computed on Cα covariance matrices within the essential-dynamics framework [30]. Free energies were obtained from the Boltzmann inversion of the joint probability over the first two components, and dynamic cross-correlation matrices were computed from mass-weighted Cα fluctuations. Conformational heterogeneity was summarized with pairwise-RMSD matrices and with kernel density estimates of the RMSD and radius-of-gyration distributions. Binding free energies were estimated by MM-GBSA as implemented in gmx_MMPBSA. Frames were extracted at 1-ns intervals after the equilibration period, giving 500 frames per system. Solvation was treated with the generalized Born model (igb = 5, mbondi2 radii); the entropy term was omitted. This approximation is reasonable in the present comparison because the three ligands originate from a single similarity series seeded on 0G6 and are closely matched in size and flexibility, each containing 29 heavy atoms, so the configurational entropy contribution is expected to be of comparable magnitude across the three complexes and to cancel substantially when they are ranked against one another. The values reported here represent relative binding energies rather than absolute binding free energies, and the final ranking was determined by considering both trajectory-based stability measures and MM-GBSA estimates. The binding free-energy components were decomposed into van der Waals, electrostatic, polar solvation, and non-polar solvation terms to characterize the energetic contributions governing the interaction of each compound with the protein. Trajectory coordinates were recorded at 100-ps intervals, and the time-series profiles were smoothed using a 0.5-ns rolling mean for visualization purposes. This smoothing was applied only to the displayed time-series data and did not alter the underlying trajectory data. All reported mean values, standard deviations, and statistical analyses were calculated using the original unsmoothed per-frame data.

## 3. Results

### 3.1. Transcriptomic target prioritization identifies GZMA as the top candidate

Quality control removed 5 of the 42 samples in GSE26415. Two were excluded at the array-level screen: GSM648422, flagged only by deviation of its Spearman correlation (mean rho = 0.921 against a lower fence of 0.931), and GSM648424, flagged only by Mahalanobis distance on the first two principal components (D2 = 7.37 against a chi-square cutoff of 5.99); the median-intensity interquartile-range criterion flagged neither. Three further samples, GSM648314, GSM648320 and GSM648412, fell outside the 95 percent confidence ellipse after surrogate-variable correction and were also removed. The five excluded samples comprised three ASD cases and two controls, a distribution independent of case status (Fisher exact test, p = 1.00), leaving 18 ASD cases and 19 controls for differential expression (Supplementary Fig S1-A in S1 File). Vooma-weighted limma modeling identified 326 differentially expressed genes at a false discovery rate below 0.05 and an absolute log2 fold change of at least 0.65. After removal of unmapped probes, 223 genes remained, and a further 37 generic structural genes were filtered, which left 186 differentially expressed genes for downstream analysis, 23 upregulated and 163 downregulated in ASD (Supplementary Fig S1-B in S1 File). The pronounced downward skew is not an artifact of the fold-change threshold, since 92.4 percent of all genes significant at a false discovery rate below 0.05 were downregulated even with no fold-change filter applied, and the median log2 fold change across all probes was −0.13, which argues against a global scaling or normalization artifact. Surrogate-variable analysis identified and adjusted for one latent covariate, so residual batch structure is an unlikely explanation. Cell-type deconvolution of the same samples indicates instead that the skew has a compositional basis: estimated abundances of T cells, natural killer cells and CD8 + T cells, together with the aggregate cytotoxicity score, were all significantly lower in ASD (Wilcoxon, false discovery rate below 0.05), consistent with a contracted circulating cytotoxic-lymphocyte compartment that lowers the measured abundance of transcripts expressed predominantly in those cells. Enrichment of this set was dominated by immune effector terms, in particular, immune effector process, leukocyte and lymphocyte-mediated immunity, regulation of natural killer cell-mediated cytotoxicity and cell killing, cytokine-cytokine receptor interaction, and antigen processing and presentation (Supplementary Fig S2 in S1 File).

The composite prioritization score, which combined interaction degree, interaction betweenness, and differential-expression magnitude, placed GZMA first among all candidates, ahead of KLRK1 and KLRB1 (Table 1). Weighted co-expression analysis identified a module correlated with ASD status (r = −0.47, p = 0.003), and GZMA served as a hub within it (Supplementary Figs S3A-B in S1 File). The Drug-Gene Interaction Database returned no approved small-molecule inhibitor of GZMA, confirming that the target is currently undrugged and strengthening the case for a virtual-screening campaign.

**Table 1:** Top candidate drug targets ranked by composite prioritization score.

Rank	Gene	Degree	Betweenness	|log2FC|	Composite
1	GZMA	8	0.0639	0.74	0.8332
2	KLRK1	7	0.0533	0.739	0.7254
3	KLRB1	5	0.0184	0.774	0.4627
4	GZMK	6	0.0073	0.663	0.4208
5	SNRPG	4	0.0189	0.768	0.4054
6	KLRF1	3	0.0171	0.908	0.3987
7	ITGA2B	3	0.0024	1.016	0.3758
8	KLRC3	4	0.0061	0.831	0.3718
9	POLR2K	3	0.0159	0.733	0.3189
10	FCER1A	1	0	1.133	0.3

Columns show interaction degree, betweenness centrality, absolute log2 fold change, and the normalized composite score. GZMA ranks first across the weighted metrics. Numeric cells are to be completed from the network output.

### 3.2. Virtual screening yields three drug-like GZMA candidates

Docking of the 5,309 ZINC compounds against the GZMA active site returned binding affinities from −4.4 to −9.3 kcal/mol. The three compounds carried into simulation, T1, T2, and T3, scored −9.3, −9.1, and −8.9 kcal/mol, respectively, against −7.4 kcal/mol for the co-crystal ligand 0G6 under identical conditions (Table 2). All three poses sat within the S1 and S2 subsites and formed at least one direct hydrogen bond to a catalytic or substrate-binding residue, with T1 contacting the catalytic His57 through two salt bridges in addition to four hydrogen bonds (Fig 1). The reference pose reproduced the contacts expected from the co-crystal complex, including Arg99, Ser190, Ser195, Phe215, Gly216, and Glu218.

**Table 2:** Docking scores and interaction profiles of the three top hits and the reference ligand 0G6.

Cmpd	ZINC ID	Vina	Hb	HPh	pi-S	SB	Hg	Interacting residues
Ref	0G6 (Reference)	−7.4	6	4	1	–	–	Arg99, Pro174, Ser190, Asn192, Ser195, Phe215, Gly216, Glu218
T1	ZINC26097238	−9.3	4	4	–	2	–	His57, Arg99, Pro174, Ser190, Ser195, Thr213, Phe215, Gly216
T2	ZINC7143246	−9.1	3	2	–	–	1	Ser190, Ser195, Phe215, Glu218
T3	ZINC7143248	−8.9	3	2	–	–	1	Ser190, Ser195, Ser214, Phe215, Glu218

Hb, hydrogen bonds; HPh, hydrophobic contacts; pi-S, pi-stacking; SB, salt bridge; Hg, halogen bond.

**Fig 1:**
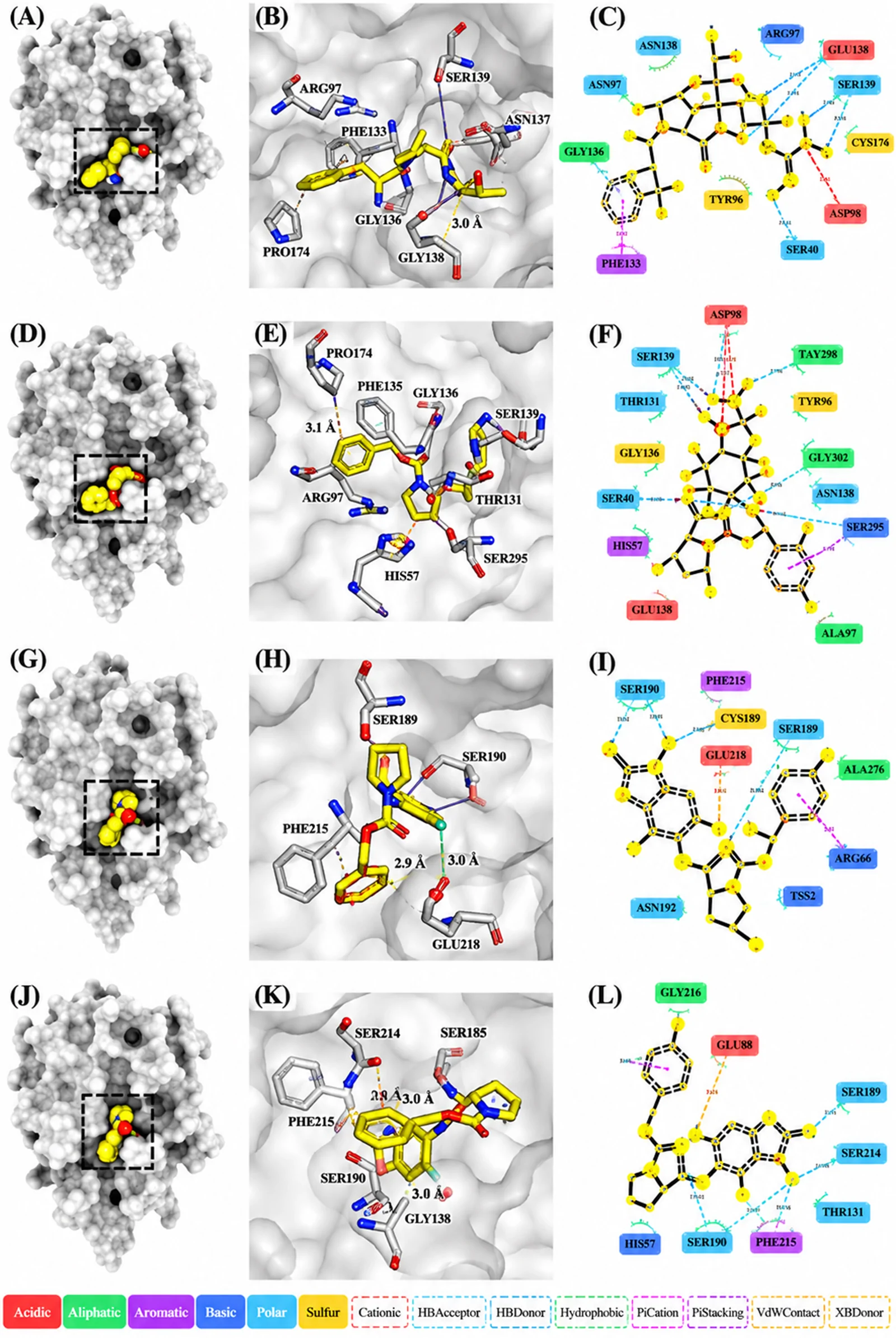
Molecular docking and binding-pose visualization of the reference inhibitor (Ref) and the candidate ligands (T1, T2, T3) in the catalytic active site of GZMA. Left column, surface representation of the enzyme with each ligand seated in the catalytic cleft. Middle column, three-dimensional view of the docked ligand with key active-site residues, where dashed lines mark hydrogen bonds and electrostatic contacts. Right column, two-dimensional interaction diagrams with residues color-coded by physicochemical class. This panel establishes the qualitative outcome confirmed by the remainder of the analysis: T2 was a stable binder, T1 was metastable, and T3 did not hold its pose.

### 3.3. Dynamic stability analysis through RMSD

Backbone RMSD stabilized within the first 50 ns in all four systems and then fluctuated inside a narrow window of roughly 1.0 to 2.5 A for the rest of the run (Fig 2A). No system showed global unfolding. The apo enzyme drifted upward over the final third of the trajectory toward about 2.5 A after 400 ns, consistent with a less constrained unliganded protein. The three complexes were all at intermediate values, and T1 and T3 were in the apo range late in the run. The pose-specific panels were more representative of binding quality. Ligand RMSD fitted on the ligand, which reports internal flexibility, settled at a plateau around 2.3 A for T2 and also varies more widely for T1 and T3 (Fig 2B).

**Fig 2:**
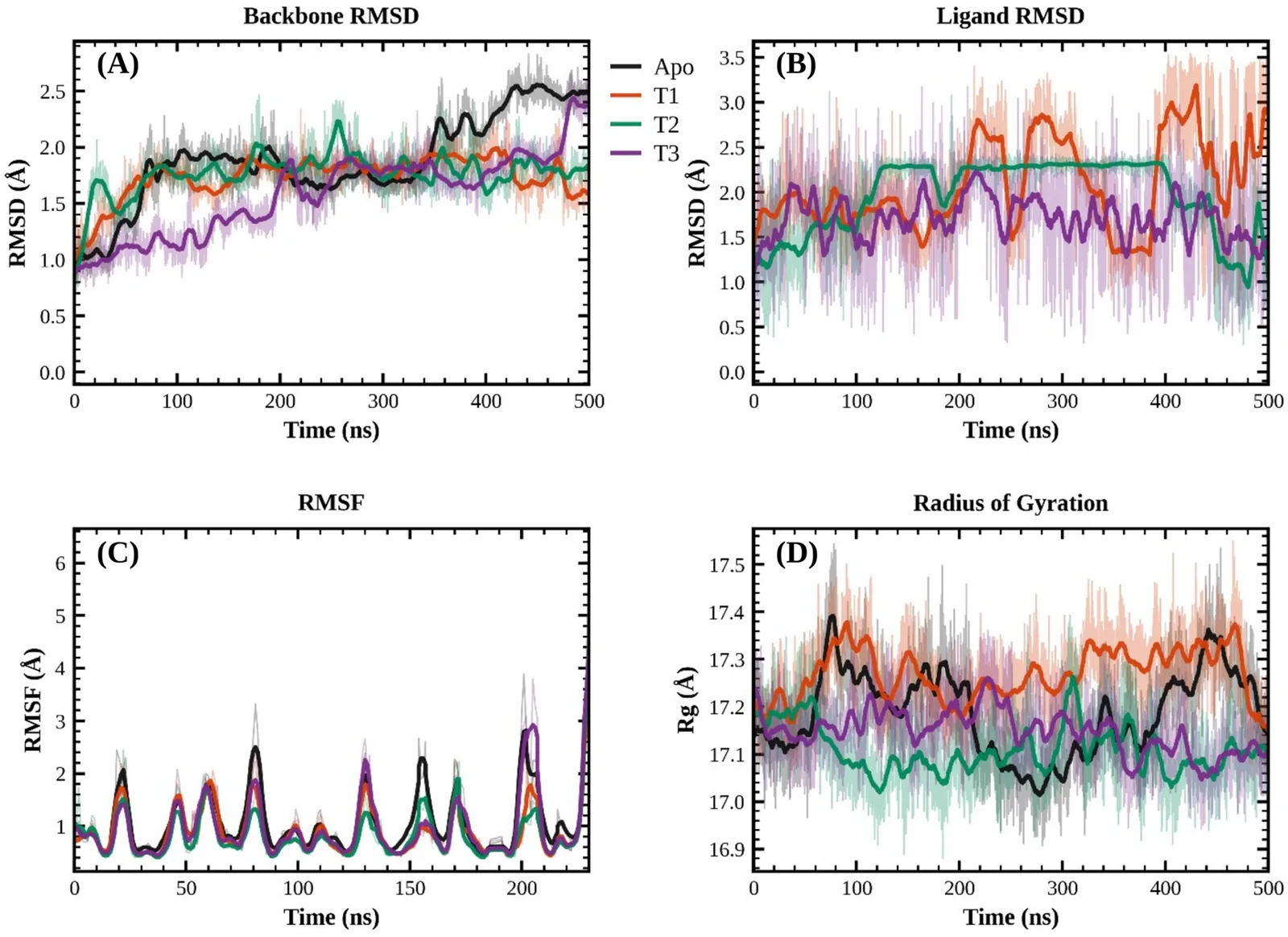
Structural stability over 500 ns. (A) Backbone RMSD for all systems. (B) Ligand RMSD. (C) Per-residue RMSF. (D) Radius of gyration. Trajectories are shown with a centered 0.5 ns rolling average applied to the raw 100 ps-interval frames for display only.

### 3.4. Flexibility analysis

Per-residue RMSF profiles shared a common shape across systems, with rigid beta-strand cores near 0.4 to 0.7 A, more mobile surface loops, and a highly mobile chain terminus where the final residues reached 5–6 A (Fig 2C). Three regions distinguished the trajectories. The loop near residue 97 was substantially more flexible in the apo enzyme, peaking near 3.3 A, than in any liganded system, demonstrating that occupancy of the catalytic cleft damps motion of a nearby loop. The region near residues 170–190 was again most mobile in the apo protein. The C-terminal segment near residues 218–225 was elevated specifically in T3, reaching about 3.8 A, in keeping with the local destabilization that accompanies its escape from the pocket. Across the panel, ligand binding reduced flexibility in the catalytic-cleft loops relative to the apo state, with the effect weakest, with a local reversal near the C-terminus, for T3.

### 3.5. Structural compactness analysis

The radius of gyration remained between 17.0 and 17.5 A in all systems, and no expansion or collapse occurred (Fig 2D). The differences were small but easy to detect. T2 was the most compact ensemble in all and had a modal value of around 17.05 to 17.15 A. T3 was slightly more compact at 17.1 to 17.2 A. T1 was the most expanded at around 17.25 to 17.35 A, and the apo protein was larger in the first 120 ns. The compaction of T2 is consistent with a ligand that binds well inside the pocket and couples the two beta barrel lobes, while the expansion of the T1 ensemble is consistent with the looser complex inferred from its pose RMSD.

### 3.6. Hydrogen bonding analysis

In all systems, intra-protein hydrogen bonds were largely conserved (150–165 per frame), with only the apo protein dropping modestly to ∼ 140–150 in the second half of the run, which is consistent with the higher RMSD and radius of gyration (Fig 3, left). The number of protein ligands was far more discriminating (Fig 3, right). T2 maintained a steady 2–3 bonds for the entire run. T1 was intermittent and formed bonds in the middle of the run before they collapsed as the ligand left the site. T3 formed almost no persistent bonds beyond about 50 ns [22].

**Fig 3:**
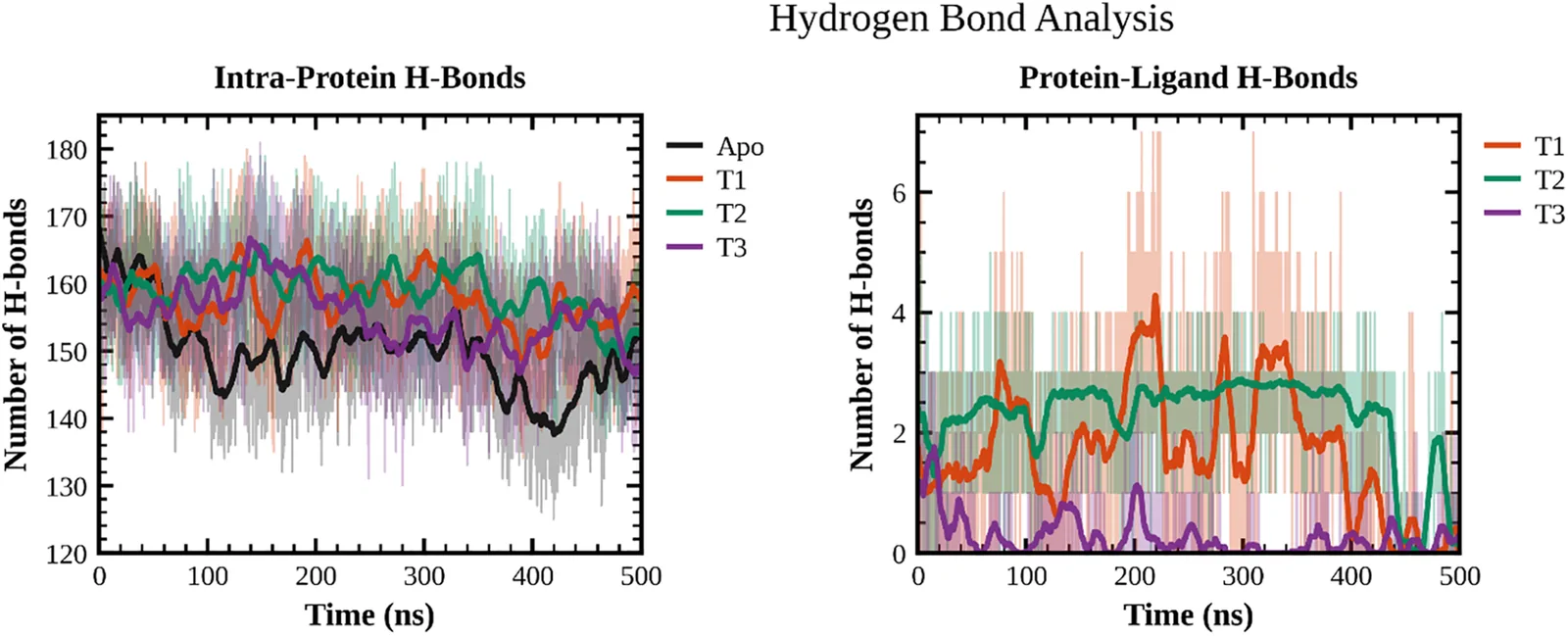
Hydrogen-bond analysis. Left, intra-protein bonds. Right, protein-ligand bonds. Both shown with a centered 0.5 ns rolling average applied to the raw 100 ps-interval frames for display only.

### 3.7. Short-range interaction energies favor T2

Short-range Lennard-Jones and Coulomb energies decomposed the enthalpic part of the interaction into shape-complementary van der Waals and electrostatic contributions (Fig 4). T1 achieved strong van der Waals packing early but consistently weaker electrostatics near −40 to −75 kJ/mol, and both terms decayed toward zero as it dissociated. T2 showed moderate but durable interactions in both terms, with Lennard-Jones near −100 to −125 kJ/mol and Coulomb near −75 to −100 kJ/mol. T3 had the weakest and most intermittent energies, with both terms repeatedly returning to about zero, the signature of a ligand that spends much of the run out of contact. The contrast between T1 and T2 was informative: T1 appeared to be held mainly by transient hydrophobic packing that did not survive once the complex loosened, whereas T2 combined van der Waals and electrostatic anchoring that resisted dissociation.

**Fig 4:**
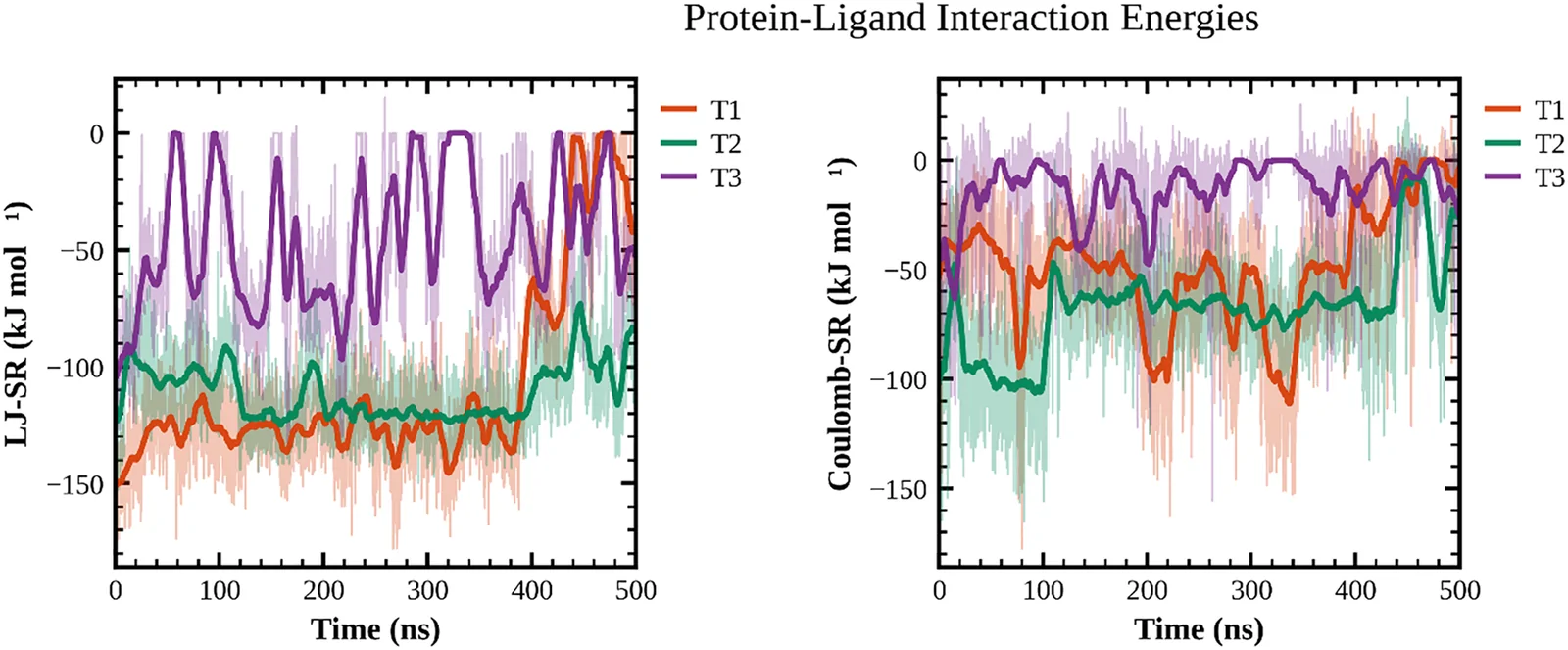
Short-range protein-ligand interaction energies. Left, Lennard-Jones. Right, Coulomb.

### 3.8. Binding-pocket occupancy confirms the ranking

Pocket-specific descriptors indicate how well each ligand has occupied the catalytic cleft over time (Fig 5). Contacts within 0.35 nm were clearly separated: T1 was 100–150 before rapidly falling away from it after about 400 ns, T2 was 60–80, and T3 lost virtually all contact within 50 ns. The same phenomenon was observed for pocket hydrogen bonds. The trajectory-averaged values for T1 and T2 were quantified as well (Table 3): mean contacts of 106.1, 68.8, 5.4, and mean pocket hydrogen bonds of 1.7, 2.3, and 0.3. The large standard deviation on the T1 contact is representative of the bound-then-unbound nature of T1, and the small dispersion on T2 is the result of the pocket being continuously occupied. T1 exhibited higher mean contacts than T2 but formed fewer hydrogen bonds and did not remain bound. Its greater mean contact count is a matter of the transient bound states before dissociation (when the ligand sat deep in the cleft) and not of superior binding quality, as the mean follows a trajectory that ends in unbinding. This is why the pose-RMSD and energy data were used to interpret the contact counts correctly.

**Table 3:** Trajectory-averaged binding-pocket metrics (mean).

Ligand	Mean pocket contacts (< 0.35 nm)	Mean pocket H-bonds	Pose stability over 500 ns
T1	106.1	1.7	Metastable, dissociates near 400–430 ns
T2	68.8	2.3	Stable, bound throughout
T3	5.4	0.3	Unstable, escapes near 50 ns

**Fig 5:**
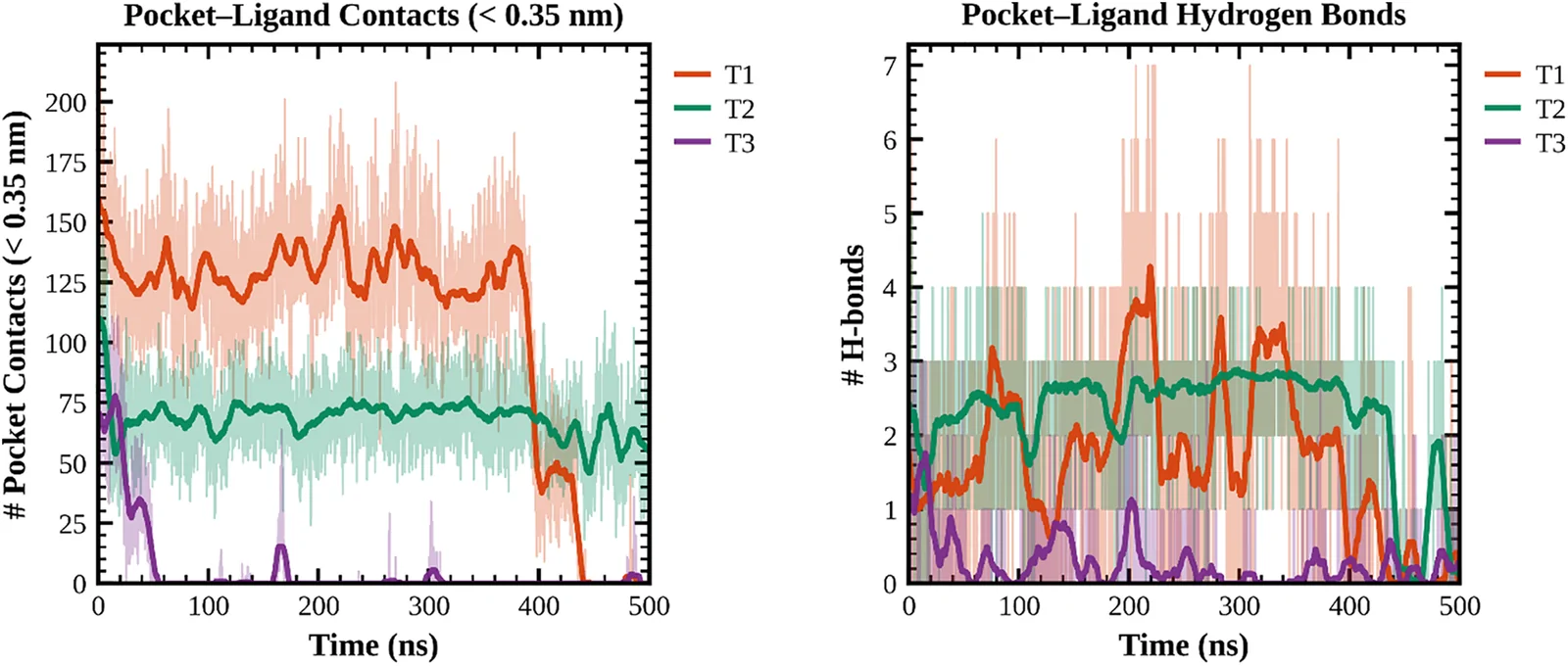
Binding pocket-ligand interactions. Left, Pocket contacts within 0.35 nm over time. Right, Pocket hydrogen bonds over time.

### 3.9. PCA and free energy surfaces reveal distinct conformational basins

Principal component analysis reduced backbone motion to the few collective coordinates that carried most of the variance [30]. Projection onto the first two components, colored by simulation progress, showed that the apo enzyme sampled the broadest space and migrated across several clusters from start to end, as expected for an unconstrained protein exploring substates (Fig 6). T2 showed a focused distribution with start and end points close together. T1, and more strongly T3, displayed separated clusters with start and end points in different basins, the signature of a discrete conformational transition, which for T3 coincides with ligand escape. The free energy surfaces projected onto the first two components made these differences quantitative (Fig 7). T2 presented a single broad, well-defined global minimum, a funnel-like landscape typical of a conformationally settled complex. The apo enzyme and T1 showed rugged surfaces with several comparable minima separated by low barriers. T3 was bistable, with two deep, well-separated minima, the energetic counterpart of the two-state behavior seen in its pose RMSD and pairwise-RMSD map. The pairwise-RMSD matrices gave a model-free view of the same pattern: the apo enzyme and the test complexes showed several blocks, and T3 displayed a striking two-block organization with a transition near 200 ns (Supplementary Fig S4 in S1 File). Taken together, these analyses reveal a coherent picture. Ligand binding restricted and smoothed the conformational landscape in proportion to binding quality, strongest for T2 and weakest for T3, and the poor binders pushed the enzyme toward multi-basin or bistable behavior.

**Fig 6:**
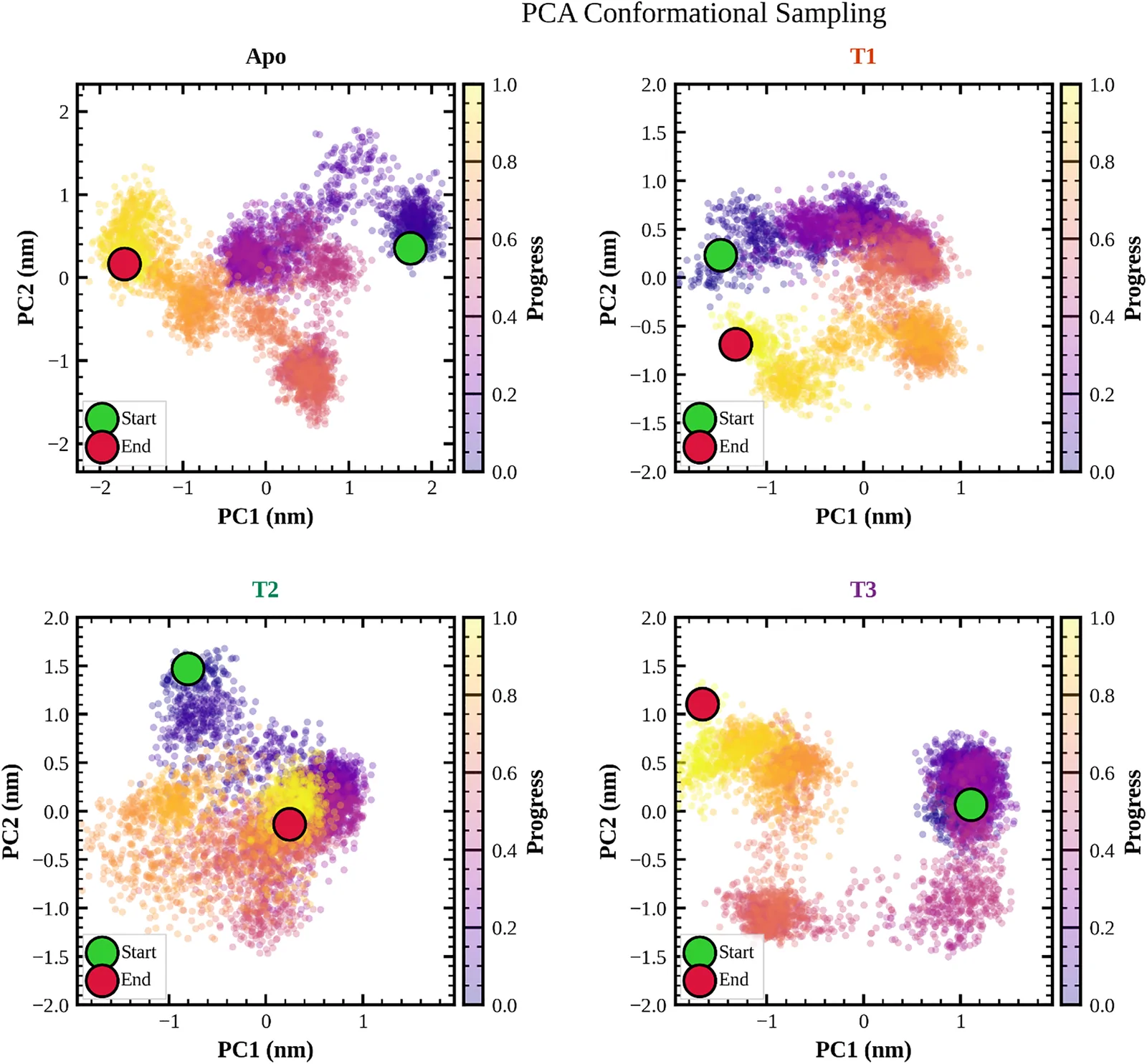
PCA conformational sampling. Scatter of PC1 against PC2 for each system, colored by simulation time, with start and end frames marked.

**Fig 7:**
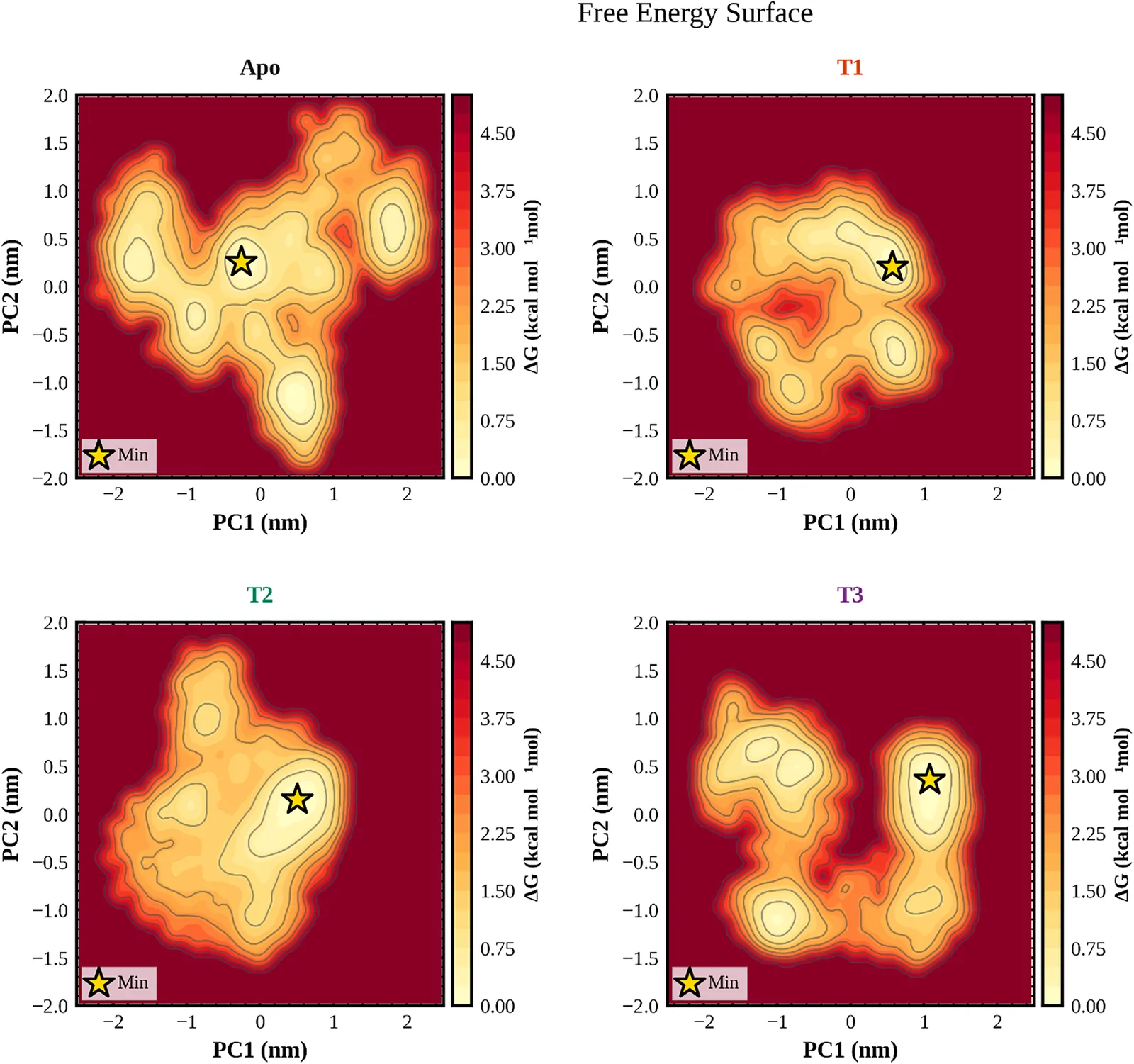
Free energy surfaces projected onto PC1 and PC2. Contours at 0.5 kcal/mol intervals; the star marks the global minimum.

### 3.10. Dynamic cross-correlation reveals ligand-specific coupling

Dynamic cross-correlation matrices report whether residue pairs move together or in opposition and are used to detect ligand-induced changes in a protein's internal coupling (Supplementary Fig S5 in S1 File). All systems showed strong positive correlation along the diagonal and within contiguous secondary-structure elements. The off-diagonal pattern was ligand-dependent. T1 showed comparatively organized correlated blocks with muted anti-correlation, suggesting that an occupied pocket couples the dynamics of the two lobes into a more concerted motion. The apo enzyme showed pronounced anti-correlated patches between the catalytic-cleft loops and distal regions, consistent with freer, more independent domain motion. T2 produced a weaker, more diffuse pattern, pointing to partial decoupling of inter-lobe motion. T3 stood apart, with correlation and anti-correlation rearranged in the C-terminal region near residues 185–225, the same segment flagged as hyper-flexible by RMSF, implying that loss of the ligand reorganized the dynamic network rather than only perturbing it locally.

### 3.11. Probability-density analysis

Kernel density estimates of RMSD, and radius of gyration summarized the ensembles compactly (Fig 8). The RMSD distribution showed a sharp peak for T2 near 1.8 A, indicating a single dominant state. The apo distribution was bimodal, with a major peak near 1.4 A and a second population extending toward 2.5 A, direct evidence of two-state behavior in the unliganded enzyme. T3 was broad and multimodal, spanning roughly 1.1 to 2.4 A. The radius-of-gyration distribution repeated the compactness ranking, with T2 most compact and T1 most expanded, and the joint RMSD to Rg density confirmed that each system occupied a largely non-overlapping region of the structural plane. The narrowness of the T2 density against the breadth of the apo and T3 densities provided an ensemble-level confirmation of the time-series conclusions.

**Fig 8:**
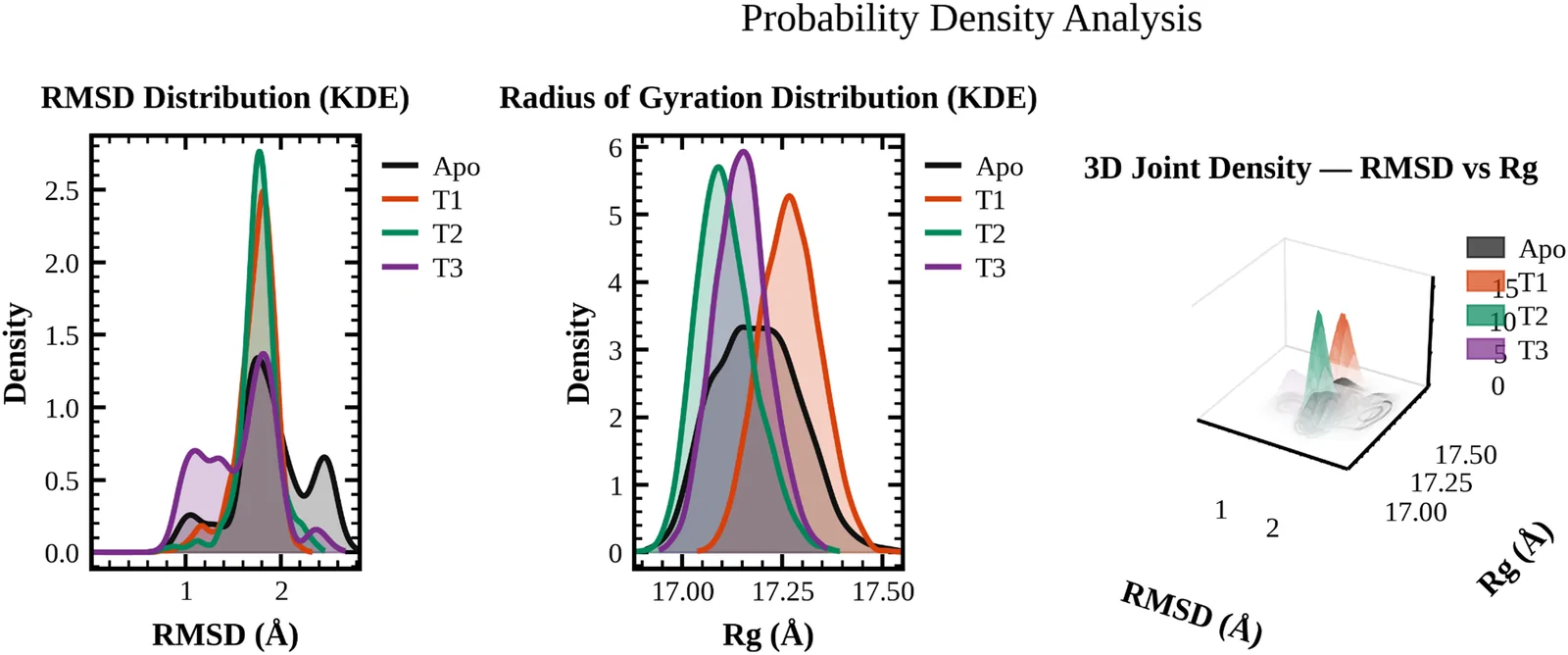
Probability-density analysis. Left, RMSD distribution. Center, radius-of-gyration distribution. Right, joint RMSD to Rg density.

### 3.12. MMGBSA analysis

The binding free energies (ΔGbind) and their individual energetic components for the three ligands (T1, T2, and T3) were calculated using the MM/GBSA method to quantitatively evaluate their respective binding affinities (Table 4). Among the evaluated systems, ligand T2 demonstrated the most favorable binding affinity, possessing the lowest total binding free energy of −23.90 ± 5.03 kcal/mol. Ligand T1 exhibited a slightly weaker but comparable affinity with a ΔGbind of −20.49 ± 9.49 kcal/mol, whereas ligand T3 showed a substantially weaker binding profile at −8.49 ± 7.12 kcal/mol, indicating significantly lower stability in the binding pocket. An assessment of the individual thermodynamic components reveals that van der Waals interactions (ΔEvdW) were the dominant favorable contributor to the overall binding free energy across all complexes. Ligand T2 displayed the strongest van der Waals interactions at −35.70 ± 2.52 kcal/mol, closely followed by T1 (−33.00 ± 4.75 kcal/mol) and T3 (−21.50 ± 3.56 kcal/mol). Similarly, electrostatic interactions (ΔEele) favorably contributed to binding, with T2 again exhibiting the highest stabilization (−13.10 ± 2.52 kcal/mol) compared to T1 (−11.40 ± 4.75 kcal/mol) and T3 (−6.20 ± 3.56 kcal/mol). As expected for protein-ligand binding, the polar solvation energy (ΔGpol) acted as the primary unfavorable component, heavily opposing binding in all three complexes with values of 27.80 ± 4.75 kcal/mol for T1, 29.00 ± 2.52 kcal/mol for T2, and 21.40 ± 3.56 kcal/mol for T3. However, this penalty was partially offset by minor favorable contributions from non-polar solvation energies (ΔGnp), which were −4.40 ± 4.75 kcal/mol for T1, −4.60 ± 2.52 kcal/mol for T2, and −3.00 ± 3.56 kcal/mol for T3. In summary, the formation of these ligand complexes is primarily driven by strong favorable van der Waals interactions and secondary electrostatic forces, which together successfully overcome the unfavorable polar solvation penalties to establish the superior binding affinity of T2.

**Table 4:** MM-GBSA Binding Free Energy Components (kcal mol^−1^).

Ligand	ΔEvdW(mean±SD)	ΔEele(mean±SD)	ΔGpol(mean±SD)	ΔGnp(mean±SD)	ΔGbind(mean±SD)
T2	−35.70 ± 2.52	−13.10 ± 2.52	29.00 ± 2.52	−4.60 ± 2.52	**−23.90 ± 5.03**
T1	−33.00 ± 4.75	−11.40 ± 4.75	27.80 ± 4.75	−4.40 ± 4.75	**−20.49 ± 9.49**
T3	−21.50 ± 3.56	−6.20 ± 3.56	21.40 ± 3.56	−3.00 ± 3.56	**−8.49 ± 7.12**

Values in kcal mol^−1^. ΔEvdW = van der Waals; ΔEele = electrostatic; ΔGpol = polar solvation (GB); ΔGnp = non-polar solvation (SASA); ΔGbind = total binding free energy.

## 4. Discussion

The analysis provided a consistent overview of how the three ligands bound to Granzyme A compared with the unliganded apo enzyme. The apo protein showed the greatest conformational flexibility of any system, sampling the largest conformational space and showing a shift towards higher backbone RMSD and radius of gyration over the simulation. It also provided a rugged free energy landscape with multiple comparable minima rather than a single well. This behavior provides the baseline for the test compounds, and the departure of the liganded systems from this baseline supports the docking and simulation procedure, as a stable binder is expected to remain associated with the binding site and potentially restrict enzyme dynamics relative to the apo state [6].

The most promising of the three test compounds is T2. It makes fewer pocket contacts than T1 (68.8 against 106.1) but remains bound for more than 500 ns, is more stable in hydrogen bonds than T1, has balanced van der Waals and electrostatic interactions, and produces a single minimum free energy surface that is characteristic of a conformationally stable complex. T2 is a genuine, although lower-affinity, binder whose complex is well behaved and provides a favorable profile for lead development. T1 highlights why docking scores alone cannot establish binding stability. It has the most favorable docking affinity of the set at −9.3 kcal/mol, is deepest in the cleft, and even reaches the catalytic His57 through two salt bridges. In the dynamics, it strongly engaged the pocket for the first 400 ns with strong van der Waals packing, but its weak electrostatic complementarity and the instability of hydrogen bonding made it vulnerable, and it dissociated from the binding site during the last 70–100 ns. Its large variation in the pocket-contact statistic and its large radius of gyration and multi-minimum landscape all suggest the transition. The top-ranked docking pose was the least stable of the two stable-looking candidates, whereas the second-ranked compound, T2, was the one that remained stable throughout the simulation. The most logical step is to strengthen polar contacts to the catalytic cleft and increase the residence time of T1. Such approaches have been widely used to study the structural mechanism of different drugs against different targets in different diseases [31–33]. T3 is not a good binder since it dissociated from the binding pocket in about 50 ns, formed almost no persistent contacts or hydrogen bonds, had almost zero interaction energies for most of the run, and drove the enzyme into a clearly bistable state with two deep free energy minima, a two-block pairwise-RMSD map, an early principal component transition, and a reorganized C-terminal network. Its docking pose was not retained in the dynamics and is best considered a docking artifact. The gap between a plausible docking score of −8.9 kcal/mol and the rapid dissociation in solution provides a clear reason to place molecular dynamics as a post-docking stability filter.

The thermodynamic profiling of MM/GBSA further explains these different dynamics. The docking scores in the first place were very favorable to T1, but the endpoint free energy calculations for T2 further demonstrate that T2 is the most thermodynamically stable complex. The better binding affinity of T2 is not just due to the total number of contacts, but to the excellent van der Waals interactions that effectively reduce the large polar desolvation penalty of the binding cleft. This strong non-covalent packing may explain why T2 maintains a single minimum free energy surface and remains anchored for the full 500 ns.

T1 outscores T2 on raw mean contacts yet is the less reliable binder, and the apo enzyme looks stable in backbone RMSD while concealing a two-state ensemble that only the RMSD density and PCA reveal. The complementary descriptors used here, pose RMSD relative to the protein, decomposed interaction energy, free energy surfaces, and cross-correlation, are what separate persistent binding from transient occupancy. Ligand binding also rigidified the catalytic-cleft loops and coupled the inter-lobe dynamics relative to the apo enzyme, in line with the expectation that occupancy of a serine-protease active site restrains the loops that line the substrate groove [22,30].

The cross-correlation results suggest that the ligands do more than occlude the active site. The organized inter-lobe coupling under T1 and the C-terminal reorganization that follows the departure of T3 point to ligand-specific effects on the dynamic communication network. These are hypotheses rather than conclusions, and solution methods such as NMR relaxation or hydrogen-deuterium exchange would be needed to test them.

From a therapeutic standpoint, the pyroptotic pathway opened by GZMA cleavage of gasdermin B is an attractive intervention point because it acts upstream of the cytokine release that follows pore formation [7,9]. Blocking GZMA before gasdermin assembles into pores would, in principle, prevent the coordinated release of interleukin-1-beta, interleukin-18, and HMGB1 that accompanies pyroptotic death, a cleaner approach than neutralizing those mediators downstream. Direct support comes from the finding that inactivating GZMA suppresses gasdermin-B pyroptosis and limits inflammation in experimental settings [10]. Whether this axis is active in brain parenchyma or mainly in peripheral tissue remains open, but the correlation of peripheral GZMA expression with behavioral severity in the GSE26415 cohort supports at least a biomarker relationship that may index central inflammation indirectly [1]. Deconvolution of the same cohort indicates that the reduction in peripheral GZMA transcript is largely compositional rather than a reduction in per-cell expression: the group effect on GZMA fell from a coefficient of −0.89 (p = 0.003) to −0.18 (p = 0.38) once estimated natural killer and CD8 + T-cell abundances were entered as covariates, and GZMA expression tracked estimated natural killer abundance closely (Spearman rho = 0.77). This is consistent with the interpretation advanced here, in which a contracted circulating cytotoxic compartment reflects redistribution rather than suppressed granzyme expression, and it reinforces the argument that GZMA is better evaluated as enzymatic activity in tissue than as transcript abundance in blood.

Several limitations temper these conclusions. Single 500 ns replicas per system limit statistical certainty, and independent replicates with different initial velocities would be needed to establish convergence and to attach confidence intervals to the dissociation events seen for T1 and T3. The short-range interaction energies are force-field non-bonded terms rather than free energies, so absolute affinity should be estimated with alchemical free energy perturbation or an end-point method that includes a configurational-entropy correction. The small-molecule parameters were assigned by the CGenFF server, so unusual functional groups carry higher uncertainty [27]. The simulations model a non-covalent interaction, and a covalent mechanism, if present for any hit, would not be captured. The transcriptomic evidence comes from a single cohort on a single platform, and replication in an independent RNA-seq dataset would strengthen the target case. Sampling adequacy over the final 100 ns was assessed from the backbone RMSD, the principal-component projections and the pairwise-RMSD maps, none of which showed a new basin being entered late in any trajectory; the apo enzyme and T3 nevertheless retained multi-basin character throughout, so slow motions on timescales beyond the 500 ns sampled here cannot be excluded and could in principle modify the dissociation behaviour inferred for T1 and T3.

## 5. Conclusion

Integrative transcriptomic analysis coupled with a composite target-prioritization framework identified GZMA as the highest-ranking therapeutic candidate for autism spectrum disorder (ASD), outperforming other immune-associated targets including KLRK1 and KLRB1. Structure-based screening of a ZINC similarity library returned three drug-like candidates whose 500 ns trajectories were benchmarked against the apo enzyme. Among the test compounds, T2 retained its pose for the full trajectory with steady pocket occupancy and a single low-energy basin, T1 bound well for roughly 400 ns before dissociating despite the best docking score, and T3 left the pocket within 50 ns. T2 is therefore the most credible lead for experimental validation, whereas T1 is metastable, holding its pose for roughly 400 ns before dissociating, and is retained only as a secondary candidate that would require optimization of its polar contacts to the catalytic cleft to improve residence time. The work also shows, within a single target, that a top docking pose can fail under dynamics while a lower-ranked pose endures, which argues for routine use of 500 ns simulation as a post-docking filter. The next steps are enzyme-inhibition assays with a fluorogenic substrate and IC50 determination, cell-based pyroptosis readouts in NK-cell or T-cell co-cultures, and free energy perturbation calculations to guide optimization of the T2 scaffold.

## Supporting information

S1 File Supplementary Figs. This file contains Supplementary Figs S1-S5. S1 Fig, principal component analysis (PCA) of sample groups after SVA noise removal and differential gene expression between control and diseased samples. S2 Fig, GO biological process and KEGG pathway enrichment dot plots for the differentially expressed genes. S3 Fig, WGCNA gene network construction, module identification and module-trait correlations. S4 Fig, pairwise root-mean-square deviation (RMSD) correlation matrices for the four simulated systems. S5 Fig, dynamic cross-correlation matrices (DCCM) of residue fluctuations during the molecular dynamics simulations.
(DOCX)
