# Supplementary material for: Structure-based discovery of a novel Granzyme A (GZMA) inhibitor for autism spectrum disorder through integrative transcriptomics, virtual screening, and long-timescale molecular dynamics simulations
Source: PLoS One. 2026 Oct 13;21(10):e0359103. doi: 10.1371/journal.pone.0359103 (PMC13660882; doi:10.1371/journal.pone.0359103)
Supplement: Supplementary file 1 [file pone.0359103.s001.docx]

**Structure-Based Discovery of a Novel Granzyme A (GZMA) Inhibitor for Autism Spectrum Disorder Through Integrative Transcriptomics, Virtual Screening, and Long-Timescale Molecular Dynamics Simulations**

Metab Alharbi¹, Abdulrahman Alshammari¹, Othman Al Shabanah¹, Mohammed Mufadhe Alanazi¹, Ahmed Altuwaijri¹, Abdullah F. Alasmari¹, Talal Alshammari²

¹ Department of Pharmacology and Toxicology, College of Pharmacy, King Saud University, P.O. Box 2457, Riyadh 11451, Saudi Arabia.

² College of Pharmacy, King Saud University, Riyadh 11451, Saudi Arabia

### Email Addresses

Metab Alharbi – [mesalharbi@ksu.edu.sa](mailto:mesalharbi@ksu.edu.sa)
Abdulrahman Alshammari – [abdalshammari@ksu.edu.sa](mailto:abdalshammari@ksu.edu.sa)
Othman Al Shabanah – [shabanah@ksu.edu.sa](mailto:shabanah@ksu.edu.sa)
Mohammed Mufadhe Alanazi – [momalanazi@ksu.edu.sa](mailto:momalanazi@ksu.edu.sa)
Ahmed Altuwaijri – [aaltuwaijri@ksu.edu.sa](mailto:aaltuwaijri@ksu.edu.sa)
Abdullah F. Alasmari – [afalasmari@ksu.edu.sa](mailto:afalasmari@ksu.edu.sa)
Talal Alshammari – [444105603@student.ksu.edu.sa](mailto:444105603@student.ksu.edu.sa)

**Corresponding Author**

Metab Alharbi – [mesalharbi@ksu.edu.sa](mailto:mesalharbi@ksu.edu.sa)

Department of Pharmacology and Toxicology, College of Pharmacy, King Saud University, P.O. Box 2457, Riyadh 11451, Saudi Arabia.

**(B)**

**(A)**

**Supplementary Figure S1: Principal Component Analysis (PCA) and differential gene expression.** (A) PCA of sample groups (Control: blue; Diseased: red) after SVA noise removal, showing clustering along PC1 (20.8% variance) and PC2 (13.2% variance) with 95% confidence ellipses. (B) Volcano plot highlighting significant up-regulated (n=32, red) and down-regulated (n=294, blue) genes based on defined log2 fold change and -log_10_(P_adj_) thresholds. Grey dots represent non-significant genes (n=18,868)

**(B)**

**(A)**

**Supplementary Figure S2**. **GO Biological Process and KEGG Pathway enrichment. Dot plots showing the most significantly enriched** (A) GO Biological Process terms and (B) KEGG pathways for the identified differentially expressed genes. Dot size indicates the number of mapped genes for each term, while the color gradient (blue to red) represents the adjusted p-value (p.adjust). The x-axis indicates the Gene Ratio.

**(B)**

**(A)**

**Supplementary Figure S3. WGCNA gene network construction and module-trait correlations.** (A) Gene dendrogram obtained via average linkage hierarchical clustering, with the color bar below representing module assignments for individual genes. (B) Heatmap showing the relationship between module eigengenes and Disease Status. Cells display the correlation coefficient and p-value (in parentheses), colored according to the scale bar on the right (blue denotes negative correlation; red denotes positive correlation).

**Supplementary Figure S4. Pairwise Root-Mean-Square Deviation (RMSD) correlation matrices.** The matrices display the structural similarity and dynamic behavior for four different simulation conditions. Each matrix is a pairwise plot comparing the backbone conformations at all time points of the 500 ns simulations on both axes. The simulation time from 0 to 500 ns is plotted on both the x- and y-axes. The color scale on the right of each panel represents the RMSD in Angstroms (Å). Light blue and white colors indicate low RMSD (high structural similarity), while dark blue colors correspond to high RMSD (greater structural difference). Light-colored blocks along the diagonal reveal periods of conformational stability or meta-stable states, where the structure remains similar over a time interval.

**Supplementary Figure S5**. **Dynamic Cross-Correlation Matrices (DCCM) of residue fluctuations during the MD simulations.** The matrices illustrate the correlated and anti-correlated motions between protein residues for the Apo state, T1, T2 and T3 systems. Both the x- and y-axes represent the residue index, spanning from residue 1 to 231. The color bar indicates the cross-correlation coefficient, ranging from -1.0 to 1.0. Dark red regions denote strong positive correlation, indicating that the corresponding residues are moving in the same direction (concerted motion). Conversely, dark blue regions signify strong negative correlation (anti-correlated motion), where residues move in opposite directions. White areas indicate independent, uncorrelated movements. The continuous dark red diagonal line represents the perfect auto-correlation of each residue with itself.
