# Supplementary material for: Structure-based discovery of a novel Granzyme A (GZMA) inhibitor for autism spectrum disorder through integrative transcriptomics, virtual screening, and long-timescale molecular dynamics simulations
Source: PLoS One. 2026 Oct 13;21(10):e0359103. doi: 10.1371/journal.pone.0359103 (PMC13660882; doi:10.1371/journal.pone.0359103)
Supplement: Peer review history

# Peer review: Decision Letter 0

**DOI:** 10.1371/journal.pone.0359103.r001

**Authors:** Faisal Ahmad

16 Aug 2026

Dear Dr. Alshammari,

Thank you for submitting your manuscript to PLOS ONE. After careful consideration, we feel that it has merit but does not fully meet PLOS ONE’s publication criteria as it currently stands. Therefore, we invite you to submit a revised version of the manuscript that addresses the points raised during the review process.

==============================

==============================

Please submit your revised manuscript by Oct 15 2026 11:59PM. If you will need more time than this to complete your revisions, please reply to this message or contact the journal office at plosone@plos.org. When you're ready to submit your revision, log on to https://www.editorialmanager.com/pone/ and select the 'Submissions Needing Revision' folder to locate your manuscript file.

Please include the following items when submitting your revised manuscript:

- A letter that responds to each point raised by the academic editor and reviewer(s). You should upload this letter as a separate file labeled 'Response to Reviewers'.
- A marked-up copy of your manuscript that highlights changes made to the original version. You should upload this as a separate file labeled 'Revised Manuscript with Track Changes'.
- An unmarked version of your revised paper without tracked changes. You should upload this as a separate file labeled 'Manuscript'.

If you would like to make changes to your financial disclosure, please include your updated statement in your cover letter. Guidelines for resubmitting your figure files are available below the reviewer comments at the end of this letter.

If applicable, we recommend that you deposit your laboratory protocols in protocols.io to enhance the reproducibility of your results. Protocols.io assigns your protocol its own identifier (DOI) so that it can be cited independently in the future. For instructions see: https://journals.plos.org/plosone/s/submission-guidelines#loc-laboratory-protocols. Additionally, PLOS ONE offers an option for publishing peer-reviewed Lab Protocol articles, which describe protocols hosted on protocols.io. Read more information on sharing protocols at https://plos.org/protocols?utm_medium=editorial-email&utm_source=authorletters&utm_campaign=protocols.

As the corresponding author, your ORCID iD is verified in the submission system and will appear in the published article. PLOS supports the use of ORCID, and we encourage all coauthors to register for an ORCID iD and use it as well. Please encourage your coauthors to verify their ORCID iD within the submission system before final acceptance, as unverified ORCID iDs will not appear in the published article. *Only*  the individual author can complete the verification step; PLOS staff *cannot*  verify ORCID iDs on behalf of authors.

We look forward to receiving your revised manuscript.

Kind regards,

Faisal Ahmad

Academic Editor

PLOS One

Journal Requirements:

When submitting your revision, we need you to address these additional requirements.

1. Please ensure that your manuscript meets PLOS ONE's style requirements, including those for file naming. The PLOS ONE style templates can be found at

https://journals.plos.org/plosone/s/file?id=wjVg/PLOSOne_formatting_sample_main_body.pdf and

https://journals.plos.org/plosone/s/file?id=ba62/PLOSOne_formatting_sample_title_authors_affiliations.pdf

2. Please provide a complete Data Availability Statement in the submission form, ensuring you include all necessary access information or a reason for why you are unable to make your data freely accessible. If your research concerns only data provided within your submission, please write "All data are in the manuscript and/or supporting information files" as your Data Availability Statement.

3. PLOS requires an ORCID iD for the corresponding author in Editorial Manager on papers submitted after December 6th, 2016. Please ensure that you have an ORCID iD and that it is validated in Editorial Manager. To do this, go to ‘Update my Information’ (in the upper left-hand corner of the main menu), and click on the Fetch/Validate link next to the ORCID field. This will take you to the ORCID site and allow you to create a new iD or authenticate a pre-existing iD in Editorial Manager.

4. Please amend either the abstract on the online submission form (via Edit Submission) or the abstract in the manuscript so that they are identical.

5. Please include captions for your Supporting Information files at the end of your manuscript, and update any in-text citations to match accordingly. Please see our Supporting Information guidelines for more information: http://journals.plos.org/plosone/s/supporting-information.

6. If the reviewer comments include a recommendation to cite specific previously published works, please review and evaluate these publications to determine whether they are relevant and should be cited. There is no requirement to cite these works unless the editor has indicated otherwise.

[Note: HTML markup is below. Please do not edit.]

Reviewers' comments:

Reviewer's Responses to Questions

**Comments to the Author**

1. Is the manuscript technically sound, and do the data support the conclusions?

Reviewer #1: Yes

Reviewer #2: Yes

**********

2. Has the statistical analysis been performed appropriately and rigorously?

Reviewer #1: Yes

Reviewer #2: N/A

**********

3. Have the authors made all data underlying the findings in their manuscript fully available?

The PLOS Data policy

Reviewer #1: Yes

Reviewer #2: Yes

**********

4. Is the manuscript presented in an intelligible fashion and written in standard English?

Reviewer #1: Yes

Reviewer #2: Yes

**********

Reviewer #1: Reviewer comments

The author has provided a comprehensive analysis of “Structure-Based Discovery of a Novel Granzyme A (GZMA) Inhibitor for Autism Spectrum Disorder Through Integrative Transcriptomics, Virtual Screening, and LongTimescale Molecular Dynamics Simulations”

Minor comments

Comment 1. Please add the original Kuwano et al. citation directly after the first mention of dataset GSE26415 in the Abstract, so readers can immediately locate the primary source.

Comment 2. An absolute log₂ fold-change threshold of 0.65 (~1.57-fold) is slightly unconventional. Please provide a brief justification for this cutoff either by citing precedent in similar ASD transcriptomic studies.

Comment 3. The receptor coordinates are taken from PDB 1OPQ while the ligand reference is from PDB 1ORF. Please add one sentence clarifying why two structures were used and confirming that the active-site conformations are sufficiently similar for this cross-structure docking protocol.

Comment 4. The equilibration totals 4 ns before 500 ns production. Please confirm in the text that density and potential energy were visually monitored for convergence during this window, or state the specific criterion used to declare equilibration complete.

Comment 5. The entropy term was omitted from MM-GBSA. To substantiate the claim that this omission is negligible across similar scaffolds, please provide the approximate magnitude of the −TΔS term for one representative ligand, or cite a prior study that validates this approximation for comparable serine-protease inhibitors.

Comment 6. Two samples were removed during QC. Please specify which of the three metrics (IQR, Spearman deviation, or Mahalanobis distance) flagged them, and state whether they were from the ASD or control group, so readers can rule out case-status bias.

Comment 7. The Conclusion highlights T2 as the most credible lead but does not mention T1's metastable behavior. Please add a brief clause acknowledging T1 as a secondary candidate that would require contact optimization, consistent with the Discussion.

Reviewer #2: The manuscript entitled “Structure-Based Discovery of a Novel Granzyme A (GZMA) Inhibitor for Autism Spectrum Disorder Through Integrative Transcriptomics, Virtual Screening, and Long Timescale Molecular Dynamics Simulations “ addresses an important health concern

need by designing drugs against the Autism Spectrum Disorder. The overall strategy is logical and timely; however, the manuscript in its current form contains several technical inaccuracies, in bioinformatics, formatting errors, and generic visualization choices. Addressing the minor points below is necessary before the manuscript can be considered for publication.

• At first mention, please specify "gasdermin B (GSDMB)" to distinguish it from GSDMD, which is more commonly associated with pyroptosis in the broader immunology literature.

• The ASD prevalence statistic (1 in 31) should include the surveillance year or ADDM cycle (e.g., 2020) so readers can assess the currency of the figure, given rapidly evolving epidemiological data.

• The statement that GZMA is "the most abundant serine protease" secreted by cytotoxic lymphocytes requires a supporting citation, as granzyme B is frequently cited in that context plz check line 82.

• The DEG list is strongly asymmetric (23 upregulated vs. 163 downregulated in ASD). Please add a brief comment on whether this skew reflects a genuine biological signal or whether it might indicate a technical artifact such as batch effects or probe-bias.

• The Figure 1 caption contains panel labels "(L) (A) (B)" that appear disordered relative to the described layout. Please ensure the panel letters in the caption match the figure precisely.

• Please clarify whether the 0.5 ns rolling average was applied to the raw RMSD/RMSF trajectories or to already-binned data, as this affects how readers interpret the reported variance.

• In text citation "(Hink-Schauer et al., 2003)" appears in the Results without a corresponding numbered reference in the visible text. Please ensure all cited works are included in the final reference list.

• The text states that T1 recorded more mean contacts than T2 yet was the less reliable binder. Please clarify explicitly that T1's higher contact count reflects transient bound states before dissociation, not superior binding quality.

• Add a brief comment on whether any trajectory exhibited unsampled slow motions in the final 100 ns that might affect the dissociation conclusions.

• The text uses both "500 ns" and "half-microsecond" interchangeably. Please adopt consistent terminology throughout (preferably "500 ns") to avoid confusion.

**********

what does this mean?). If published, this will include your full peer review and any attached files.

If you choose “no”, your identity will remain anonymous but your review may still be made public.

**Do you want your identity to be public for this peer review?**  For information about this choice, including consent withdrawal, please see our Privacy Policy

Reviewer #1: No

Reviewer #2: No

**********

[NOTE: If reviewer comments were submitted as an attachment file, they will be attached to this email and accessible via the submission site. Please log into your account, locate the manuscript record, and check for the action link "View Attachments". If this link does not appear, there are no attachment files.]

To ensure your figures meet our technical requirements, please review our figure guidelines: https://journals.plos.org/plosone/s/figures

You may also use PLOS’s free figure tool, NAAS, to help you prepare publication quality figures: https://journals.plos.org/plosone/s/figures#loc-tools-for-figure-preparation.

NAAS will assess whether your figures meet our technical requirements by comparing each figure against our figure specifications.

# Author comment: Author response to Decision Letter 1

**DOI:** 10.1371/journal.pone.0359103.r002

22 Aug 2026

Response to Reviewers

Dear Editor,

We would like to submit our revised manuscript entitled “Structure-Based Discovery of a Novel Granzyme A (GZMA) Inhibitor for Autism Spectrum Disorder Through Integrative Transcriptomics, Virtual Screening, and Long-Timescale Molecular Dynamics Simulations” to your journal for possible publication. We thank both reviewers for a careful and constructive reading of the manuscript. The comments have materially improved the work.

Sincerely Yours,

Dr. Metab Alharbi

Reviewer 1

Comment 1. Please add the original Kuwano et al. citation directly after the first mention of dataset GSE26415 in the Abstract, so readers can immediately locate the primary source.

Response. Added as requested. The Abstract now attributes the dataset to its primary source at first mention, and the corresponding numbered reference (Kuwano et al., 2011) was already present in the reference list. So changes were made in the Abstract.

Comment 2. An absolute log2 fold-change threshold of 0.65 (~1.57-fold) is slightly unconventional. Please provide a brief justification for this cutoff by citing precedent in similar ASD transcriptomic studies.

Response. We agree that the value warrants justification and have added one. The cutoff corresponds to a 1.57-fold change and was adopted as a moderately conservative variant of the conventional 1.5-fold (log2 = 0.585) threshold routinely applied to whole-blood microarray data, where true effect sizes are attenuated because the measured signal is an average over a heterogeneous leukocyte population. Rather than rest the argument on precedent alone, we also verified empirically that the conclusions are insensitive to the choice. Re-running the contrast across thresholds gives:

log2FC cutoff Fold change DEGs (FDR<0.05) Up Down % down

0 (none) 1.00x 6946 530 6416 92.4%

0.585 1.50x 556 42 514 92.4%

0.65 (used) 1.57x 326 32 294 90.2%

0.75 1.68x 141 15 126 89.4%

1.00 2.00x 18 3 15 83.3%

Response. The directional composition of the DEG set is essentially invariant across this range, and GZMA (log2FC = -0.74, FDR = 0.018) is retained as significant throughout. The 0.65 threshold therefore controls the size of the list without altering its character or the identity of the prioritized target. We would be glad to add a literature precedent as well should the Editor prefer. So changes were made in Methods 2.2, and a sentence was added after the DEG definition.

Comment 3. The receptor coordinates are taken from PDB 1OPQ while the ligand reference is from PDB 1ORF. Please add one sentence clarifying why two structures were used and confirming that the active-site conformations are sufficiently similar for this cross-structure docking protocol.

Response. We are grateful for this comment, which uncovered a typographical error. The receptor structure used was PDB 1OP8, the human granzyme A structure of Hink-Schauer et al. (2003) that we cite; 1OPQ is an unrelated NMR structure of a DNA GATC site and was never used. The accession code has been corrected. We have also added the requested rationale and a quantitative check. Two structures were required because the only GZMA structure carrying a co-crystallized active-site inhibitor, 1ORF, is not the wild-type enzyme: the deposited entry is flagged as an engineered mutant and carries a Glu-for-Asn substitution at residue 157 (Asn170 of UniProt P12544). Docking against a mutant receptor was undesirable, so we took the ligand 0G6 and its reference binding pose from 1ORF, which also seeds the similarity search, and performed all docking against the wild-type receptor supplied by 1OP8, which carries no such substitution. The mutated position is remote from the catalytic machinery, lying 12.5 A from the bound ligand and 12.6 to 18.6 A from the catalytic triad, so the transferred pose is unaffected. Superposing the matched protomers confirms that the two structures are conformationally equivalent where it matters:

Region compared Atoms RMSD

Whole protomer (199 matched residues) Ca 0.60 A

Catalytic triad His57 / Asp102 / Ser195 backbone 0.34 A

Catalytic triad His57 / Asp102 / Ser195 all-atom 0.72 A

22 residues lining the 0G6 site backbone 0.44 A

22 residues lining the 0G6 site all-atom 1.04 A

Response. No active-site residue deviates by more than 1.0 A (largest, Arg99 at 0.96 A). Cross-structure docking is therefore justified. So changes were made in Methods 2.5: accession corrected to 1OP8 and a rationale with superposition statistics added.

Comment 4. The equilibration totals 4 ns before 500 ns production. Please confirm in the text that density and potential energy were visually monitored for convergence during this window, or state the specific criterion used to declare equilibration complete.

Response. Confirmed, and the description has been expanded. Equilibration comprised four successive 1 ns stages at a 1 fs time step in which harmonic position restraints were released stepwise from 400 to 50 kJ mol-1 nm-2 on backbone atoms and from 40 to 5 kJ mol-1 nm-2 on side-chain atoms, run under the NVT ensemble for the first 2 ns and the NPT ensemble (C-rescale barostat, 1 bar) thereafter, with a v-rescale thermostat at 303.15 K. Thermodynamic observables were written every 1 ps, and potential energy and system density were monitored across the window; production was begun only after both had reached stationary values with no residual drift. So changes were made in Methods 2.6; the equilibration sentence was expanded.

Comment 5. The entropy term was omitted from MM-GBSA. To substantiate the claim that this omission is negligible across similar scaffolds, please provide the approximate magnitude of the -TdS term for one representative ligand, or cite a prior study that validates this approximation for comparable serine-protease inhibitors.

Response. We have clarified the basis for the approximation and moderated the associated claim. The three ligands are not merely 'similar scaffolds' in a loose sense: they were drawn from a single similarity search seeded on the co-crystal ligand 0G6 and are closely matched in size and flexibility, each containing exactly 29 heavy atoms. The configurational entropy contribution is therefore expected to be of comparable magnitude across the three complexes and to cancel substantially when they are ranked against one another. Accordingly, the revised text describes the reported values as relative binding energies rather than absolute binding free energies, and states explicitly that the final ranking rests on the trajectory-derived stability measures in addition to the MM-GBSA estimates. We are happy to compute an interaction-entropy estimate of -TdS for T2 and add it to Table 4 if the reviewer considers it necessary. Modifications were made to Methods 2.7; the entropy justification was rewritten.

Comment 6. Two samples were removed during QC. Please specify which of the three metrics (IQR, Spearman deviation, or Mahalanobis distance) flagged them, and state whether they were from the ASD or control group, so readers can rule out case-status bias.

Response. We have provided the requested attribution, and in doing so identified and corrected an error in the reported cohort composition. Re-auditing the pipeline shows that removal occurs at two stages, not one, and that five samples in total were excluded from the 42 loaded. The manuscript previously reported only the first stage. The full attribution is:

Sample Stage Flagged by Statistic Group

GSM648422 Array-level QC Spearman deviation only mean rho = 0.921 (fence 0.931) ASD

GSM648424 Array-level QC Mahalanobis only D2 = 7.37 (chi2 cutoff 5.99) ASD

GSM648314 Post-SVA ellipse Mahalanobis (95% ellipse) D2 = 6.67 Control

GSM648320 Post-SVA ellipse Mahalanobis (95% ellipse) D2 = 10.20 Control

GSM648412 Post-SVA ellipse Mahalanobis (95% ellipse) D2 = 9.66 ASD

Response. The median-intensity interquartile-range criterion flagged no sample. The five excluded samples comprise three ASD cases and two controls, a distribution independent of case status (Fisher exact test, p = 1.00), so case-status bias can be ruled out. The analysed cohort is 18 ASD cases and 19 controls (n = 37); the manuscript previously stated 19 and 21. We emphasise that no downstream result changes, because every reported analysis was already computed on this 37-sample set: the DEG counts (326 before filtering; 186 after, 23 up and 163 down), Table 1, and all figures are unaffected. Only the descriptive sentence was incorrect, and it has been corrected. Changes were made to Results 3.1, the opening sentence rewritten; Methods 2.1 expanded to describe both screens.

Comment 7. The Conclusion highlights T2 as the most credible lead but does not mention T1's metastable behavior. Please add a brief clause acknowledging T1 as a secondary candidate that would require contact optimization, consistent with the Discussion.

Response. Added. The Conclusion now states explicitly that T1 is metastable, holding its pose for roughly 400 ns before dissociating, and is retained only as a secondary candidate that would require optimization of its polar contacts to the catalytic cleft to improve residence time. This wording matches the Discussion. Changes were made in the conclusion; the sentence on T1 was expanded.

Reviewer #2

Comment 1. At first mention, please specify “gasdermin B (GSDMB)” to distinguish it from GSDMD, which is more commonly associated with pyroptosis in the broader immunology literature.

Response. Agreed, and the distinction is worth making explicit. The abbreviation is now defined at first mention in both the Abstract and the Introduction. Changes were made in the Abstract and Introduction, paragraph 3.

Comment 2. The ASD prevalence statistic (1 in 31) should include the surveillance year or ADDM cycle (e.g., 2020) so readers can assess the currency of the figure.

Response. Added. We note for accuracy that the 1 in 31 estimate derives from the 2022 surveillance year, reported in the 2025 ADDM summary already cited as reference 1; the preceding 1 in 36 estimate is the 2020 cycle. The text now names both years so that the currency of each figure is explicit. Changes were made in the introduction, opening paragraph.

Comment 3. The statement that GZMA is “the most abundant serine protease” secreted by cytotoxic lymphocytes requires a supporting citation, as granzyme B is frequently cited in that context.

Response. The reviewer is right that the superlative is contestable, and we have moderated it rather than defend it. The sentence now reads that granzyme A is one of the most abundant serine proteases stored in the cytotoxic granules of activated T lymphocytes and natural killer cells, with a supporting citation to Kam et al. (2000), reference 26, which characterises the granzyme family. The revised claim does not depend on a ranking against granzyme B. Changes were made in the Introduction, paragraph 3.

Comment 4. The DEG list is strongly asymmetric (23 upregulated vs. 163 downregulated in ASD). Please add a brief comment on whether this skew reflects a genuine biological signal or whether it might indicate a technical artifact such as batch effects or probe-bias.

Response. This is the most substantive comment, and we have addressed it with additional analysis rather than a qualitative remark. Three lines of evidence indicate that the skew is biological. First, it is not created by the fold-change threshold: 92.4 percent of all genes significant at FDR below 0.05 are downregulated even with no fold-change filter, and the proportion is stable from 83 to 92 percent across every threshold tested (Comment 2 above). Second, it is not a global scaling or normalization artifact: the median log2 fold change across all probes is -0.13, close to zero, so the arrays are not systematically shifted. Third, surrogate-variable analysis identified and adjusted for one latent covariate, so residual batch structure is an unlikely explanation. We then tested the biological hypothesis directly by deconvolving circulating immune-cell composition from the same expression matrix (MCP-counter), which shows a contracted cytotoxic compartment in ASD:

Cell population Control (mean) ASD (mean) p (Wilcoxon) FDR

T cell 10.21 9.80 0.0045 0.027

Cytotoxicity score 9.91 9.20 0.0061 0.027

NK cell 8.35 7.91 0.0075 0.027

T cell CD8+ 12.26 11.64 0.0144 0.040

Response. Because these populations are the dominant source of the transcripts concerned, a reduction in their circulating abundance lowers the measured level of the genes they express, which accounts for the direction and the magnitude of the skew. A composition-adjusted model makes the point directly for the prioritized target: the group effect on GZMA falls from -0.89 (p = 0.003) to -0.18 (p = 0.38) once estimated NK and CD8+ T-cell abundances are included as covariates, and GZMA tracks estimated NK abundance closely (Spearman rho = 0.77). The downregulation is therefore compositional rather than a suppression of per-cell granzyme expression, which is consistent with, and strengthens, the interpretation already advanced in the manuscript that GZMA should be assessed as enzymatic activity at tissue sites rather than as transcript abundance in blood. Made changes to Methods 2.3 (deconvolution added), Results 3.1 (paragraph added), Discussion (paragraph added).

Comment 5. The Figure 1 caption contains panel labels “(L) (A) (B)” that appear disordered relative to the described layout. Please ensure the panel letters in the caption match the figure precisely.

Response. We have re-checked the caption against the figure. The panel letters the reviewer refers to were removed when Figure 1 was revised, and the caption in the present version already describes the figure by column position in the order in which the panels appear: left column, surface representation of the enzyme with the ligand in the catalytic cleft; middle column, three-dimensional view of the docked pose with active-site residues; right column, two-dimensional interaction diagram. No stray panel letters remain, and the description now corresponds to the figure layout, so no further change was required at this location. We apologise that the reviewer was working from the earlier wording. No change required; caption verified against the figure (Figure 1 caption).

Comment 6. Please clarify whether the 0.5 ns rolling average was applied to the raw RMSD/RMSF trajectories or to already-binned data, as this affects how readers interpret the reported variance.

Response. It was applied to the raw data, and we have stated this explicitly. Coordinates were written every 100 ps during production, and the displayed traces use a centered five-frame rolling mean over those raw per-frame values, which is exactly 0.5 ns. No prior binning was performed. Importantly, the smoothing is applied at the plotting stage only: all reported means, standard deviations and derived statistics, including the trajectory-averaged values in Table 3 and the MM-GBSA terms in Table 4, were computed on the unsmoothed per-frame data, so the reported variances are not deflated by smoothing. Changes were made to Methods 2.7, a sentence added; Figure 2 and Figure 3 captions were clarified.

Comment 7. In text citation “(Hink-Schauer et al., 2003)” appears in the Results without a corresponding numbered reference in the visible text. Please ensure all cited works are included in the final reference list.

Response. Corrected, and the check surfaced a second, related omission. The Results citation to Hink-Schauer et al. has been converted to the numbered style as reference 27, which was already in the list, and the author-year citation to Amadei et al. (1993) in Section 3.9 has likewise been converted to reference 25. In addition, Bell et al. (2003), the source of the 1ORF structure and of the co-crystal ligand 0G6, was cited in Methods 2.5 but had no entry in the reference list; it has now been added as reference 29. We have verified that every in-text citation now resolves to a numbered entry. Results 3.6 and 3.9; Methods 2.5; new reference 29 added.

Comment 8. The text states that T1 recorded more mean contacts than T2 yet was the less reliable binder. Please clarify explicitly that T1's higher contact count reflects transient bound states before dissociation, not superior binding quality.

Response. Stated explicitly. The revised passage notes that T1's higher mean contact count is a property of the transient bound states that preceded dissociation, during which the ligand sat deep in the cleft, and that the mean is taken over a trajectory that ends in unbinding; it is therefore not evidence of superior binding quality. Modifications were made in Results 3.8, closing sentence expanded

Comment 9. Add a brief comment on whether any trajectory exhibited unsampled slow motions in the final 100 ns that might affect the dissociation conclusions.

Response. Added to the limitations. Sampling adequacy over the final 100 ns was assessed from the backbone RMSD, the principal-component projections and the pairwise-RMSD maps, none of which showed a new basin being entered late in any trajectory. We are careful not to overstate this: the apo enzyme and T3 retained multi-basin character throughout, so motions slower than the 500 ns sampled here cannot be excluded and could in principle modify the dissociation behaviour inferred for T1 and T3. This is now stated. And added changes to the discussion, limitations paragraph.

Comment 10. The text uses both “500 ns” and “half-microsecond” interchangeably. Please adopt consistent terminology throughout (preferably “500 ns”).

Response. Done. The single occurrence of 'half-microsecond ' in the Conclusion has been replaced with '500 ns'. The manuscript now uses '500 ns' uniformly.

Attachment Submitted filename: Response_to_Reviewers.docx

# Peer review: Decision Letter 1

**DOI:** 10.1371/journal.pone.0359103.r003

**Authors:** Faisal Ahmad

10 Sep 2026

Structure-Based Discovery of a Novel Granzyme A (GZMA) Inhibitor for Autism Spectrum Disorder Through Integrative Transcriptomics, Virtual Screening, and Long-Timescale Molecular Dynamics Simulations

PONE-D-26-33794R1

Dear Dr. Alshammari,

We’re pleased to inform you that your manuscript has been judged scientifically suitable for publication and will be formally accepted for publication once it meets all outstanding technical requirements.

Within one week, you’ll receive an e-mail detailing the required amendments. When these have been addressed, you’ll receive a formal acceptance letter and your manuscript will be scheduled for publication.

An invoice will be generated when your article is formally accepted. Please note, if your institution has a publishing partnership with PLOS and your article meets the relevant criteria, all or part of your publication costs will be covered. Please make sure your user information is up-to-date by logging into Editorial Manager at Editorial Manager® and clicking the ‘Update My Information' link at the top of the page. For questions related to billing, please contact billing support.

If your institution or institutions have a press office, please notify them about your upcoming paper to help maximize its impact. If they’ll be preparing press materials, please inform our press team as soon as possible -- no later than 48 hours after receiving the formal acceptance. Your manuscript will remain under strict press embargo until 2 pm Eastern Time on the date of publication. For more information, please contact onepress@plos.org.

Kind regards,

Faisal Ahmad

Academic Editor

PLOS One

Additional Editor Comments (optional):

Reviewers' comments:

Reviewer's Responses to Questions

**Comments to the Author**

Reviewer #1: All comments have been addressed

Reviewer #2: All comments have been addressed

**********

2. Is the manuscript technically sound, and do the data support the conclusions?

Reviewer #1: Yes

Reviewer #2: Yes

**********

3. Has the statistical analysis been performed appropriately and rigorously?

Reviewer #1: Yes

Reviewer #2: N/A

**********

4. Have the authors made all data underlying the findings in their manuscript fully available?

The PLOS Data policy

Reviewer #1: Yes

Reviewer #2: Yes

**********

5. Is the manuscript presented in an intelligible fashion and written in standard English?

Reviewer #1: Yes

Reviewer #2: Yes

**********

Reviewer #1: (No Response)

Reviewer #2: (No Response)

**********

what does this mean?). If published, this will include your full peer review and any attached files.

If you choose “no”, your identity will remain anonymous but your review may still be made public.

**Do you want your identity to be public for this peer review?**  For information about this choice, including consent withdrawal, please see our Privacy Policy

Reviewer #1: No

Reviewer #2: No

**********

# Editor report: Acceptance letter

**DOI:** 10.1371/journal.pone.0359103.r004

**Authors:** Faisal Ahmad

PONE-D-26-33794R1

PLOS One

Dear Dr. Alharbi,

I'm pleased to inform you that your manuscript has been deemed suitable for publication in PLOS One. Congratulations! Your manuscript is now being handed over to our production team.

At this stage, our production department will prepare your paper for publication. This includes ensuring the following:

* All references, tables, and figures are properly cited

* All relevant supporting information is included in the manuscript submission,

* There are no issues that prevent the paper from being properly typeset

You will receive further instructions from the production team, including instructions on how to review your proof when it is ready. Please keep in mind that we are working through a large volume of accepted articles, so please give us a few days to review your paper and let you know the next and final steps.

Lastly, if your institution or institutions have a press office, please let them know about your upcoming paper now to help maximize its impact. If they'll be preparing press materials, please inform our press team within the next 48 hours. Your manuscript will remain under strict press embargo until 2 pm Eastern Time on the date of publication. For more information, please contact onepress@plos.org.

You will receive an invoice from PLOS for your publication fee after your manuscript has reached the completed accept phase. If you receive an email requesting payment before acceptance or for any other service, this may be a phishing scheme. Learn how to identify phishing emails and protect your accounts at https://explore.plos.org/phishing.

If we can help with anything else, please email us at customercare@plos.org.

Thank you for submitting your work to PLOS One and supporting open access.

Kind regards,

PLOS One Editorial Office Staff

on behalf of

Dr. Faisal Ahmad

Academic Editor

PLOS One
